# Large spin Hall magnetoresistance and its correlation to the spin-orbit torque in W/CoFeB/MgO structures

**DOI:** 10.1038/srep14668

**Published:** 2015-10-01

**Authors:** Soonha Cho, Seung-heon Chris Baek, Kyeong-Dong Lee, Younghun Jo, Byong-Guk Park

**Affiliations:** 1 Department of Materials Science and Engineering, KAIST, Daejeon 305-701, Korea; 2 Department of Electrical Engineering, KAIST, Daejeon 305-701, Korea; 3 Division of Scientific Instrumentation KBSI, Daejeon 305-806, Korea

## Abstract

The phenomena based on spin-orbit interaction in heavy metal/ferromagnet/oxide structures have been investigated extensively due to their applicability to the manipulation of the magnetization direction via the in-plane current. This implies the existence of an inverse effect, in which the conductivity in such structures should depend on the magnetization orientation. In this work, we report a systematic study of the magnetoresistance (MR) of W/CoFeB/MgO structures and its correlation with the current-induced torque to the magnetization. We observe that the MR is independent of the angle between the magnetization and current direction but is determined by the relative magnetization orientation with respect to the spin direction accumulated by the spin Hall effect, for which the symmetry is identical to that of so-called the spin Hall magnetoresistance. The MR of ~1% in W/CoFeB/MgO samples is considerably larger than those in other structures of Ta/CoFeB/MgO or Pt/Co/AlOx, which indicates a larger spin Hall angle of W. Moreover, the similar W thickness dependence of the MR and the current-induced magnetization switching efficiency demonstrates that MR in a non-magnet/ferromagnet structure can be utilized to understand other closely correlated spin-orbit coupling effects such as the inverse spin Hall effect or the spin-orbit spin transfer torques.

The spin Hall effect (SHE)^1^,^2^, the generation of a spin current from a charge current in non-magnetic (NM) materials, has drawn increasing interest because it can be utilized in spintronic devices for current-induced magnetization switching^3^,^4^,^5^ and for high speed domain wall motion^6^,^7^. In ferromagnet (FM)/NM heterostructures, the SHE induces spin accumulation at the FM/NM interfaces which interacts with the local magnetic moment in FM depending on their relative directions. The accumulated spin orientation 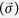, a dimensionless unit vector, is defined^1^,^2^ as


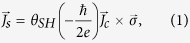


where *θ*_*SH*_ is the spin Hall angle, 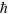 is the reduced Planck constant, *e* is th*e* elementary charge, and *J*_*s*_ (*J*_*c*_) is the spin (charge) current. Thus, spins in the *y*-direction are accumulated by spin current in the *z*-direction, which is induced by the SHE of the charge current in the *x*-direction. When 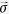 is non-collinear with regard to the magnetization direction 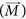, the accumulated spins are absorbed by FM, which in return exerts torque on the magnetic moment, whereas the spins are maximally reflected when 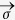 is parallel to 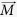. The spin current (*J*_*s*_) reflected from the FM/NM interface converts to a charge current (*J*_*c*_) via the inverse spin Hall effect (ISHE) with the same relationship expressed by Eq. (1). Since the direction of the ISHE-induced *J*_*c*_ is identical to that of the original charge current, the total current will be a sum of the two contributions, as depicted in Fig. 1(a). Consequently, the resistance of the FM/NM structures depends on the relative orientations of the magnetization and accumulated spins, which is known as the spin Hall magnetoresistance (SMR) as it is based on the SHE and on the ISHE in NM^8^,^9^,^10^. As both the SHE and ISHE rely on *θ*_*SH*_, the magnitude of SMR is proportional to the square of *θ*_*SH*_. Thus far, SMR has been investigated in FM/NM systems with a magnetic insulator, most commonly yttrium iron garnets (YIGs), which other magnetoresistance (MR) effects are absent as the current only flows through NM^8^,^10^,^11^,^12^,^13^. However, SMR should be present even in fully metallic FM/NM structures because the SHE and ISHE are generic features of NM. Note that quantitative description of the SMR in metallic structures is more complex compared to FM insulator based structures, because the current flowing through FM layer contains other MR effect such as AMR. Moreover, SMR is a closely related phenomena of the spin-orbit torque^13^, which is the spin transfer torque arising from a spin-orbit coupling effect such as the SHE and/or the interfacial Rashba effect in heavy metal/FM structures^14^,^15^,^16^,^17^,^18^,^19^,^20^. This spin-orbit torque can be utilized to manipulate the magnetization direction by an in-plane current^3^,^5^. Because the spin-orbit torques have been investigated mostly in fully metallic NM/FM structures, study on SMR in metallic structures is of great importance to understand the spin transport as well as the origin of the spin-orbit torques in such structures.

In this study, we investigate SMR in W/CoFeB/MgO structures as a function of the thicknesses of W and CoFeB layers. We observe that SMR is not sensitive to the CoFeB thickness, but is strongly dependent on the W thickness. Large SMR of about 1% is observed in W/CoFeB/MgO samples, which is nearly one order of magnitude greater than that in YIG/Pt samples^8^,^10^,^11^,^12^,^13^, and greater than those in Ta/CoFeB/MgO or Pt/Co/AlOx structures. We attribute this significant SMR in W-based structures to the large spin Hall angle of W^21^,^22^. The spin Hall angle and the spin diffusion length of W are 0.21 ± 0.01 and 2.1 ± 0.5 nm, respectively, as extracted from the thickness dependence of SMR values. Moreover, we perform spin-orbit torque-induced magnetization switching experiments and find that the trend of the switching efficiency is identical to the trend of the SMR magnitude. This confirms that the SMR and spin-orbit torques are closely correlated.

## Results

### Spin Hall magnetoresistance

**Figure 1:**
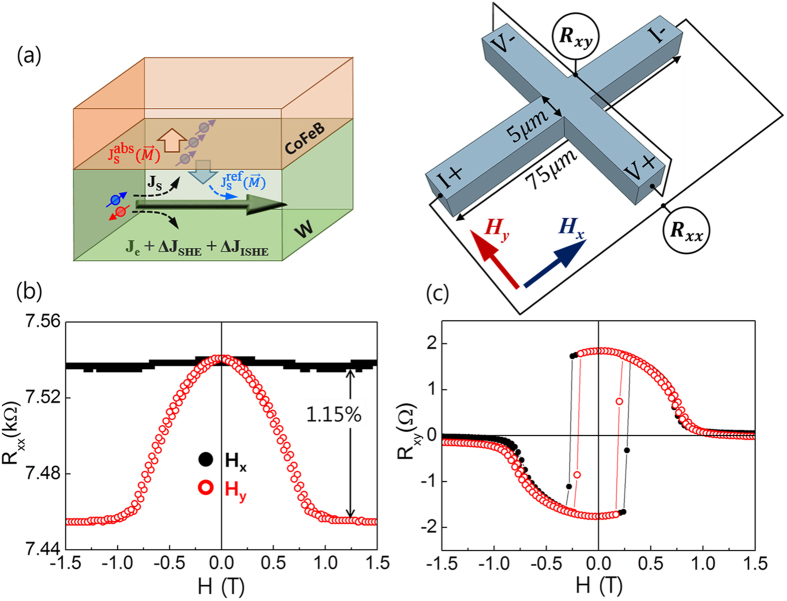
The spin Hall magnetoresistance (SMR) in W/CoFeB/MgO structure. (**a**) Schematic illustration of SMR, based on the interaction of the spin current induced by the spin Hall effect with magnetization direction. The measurement scheme of longitudinal (*R*_*xx*_) and transverse (*R*_*xy*_) resistances is shown on the right. *R*_*xx*_ (**b**) and *R*_*xy*_ (**c**) of the sample W(5 nm)/CoFeB(1.2 nm)/MgO(1.6 nm) as a function of *H*_*x*_ and *H*_*y*_, respectively. The black solid (red open) circles represent the data for *H*_*x*_ (*H*_*y*_).

**Figure 2:**
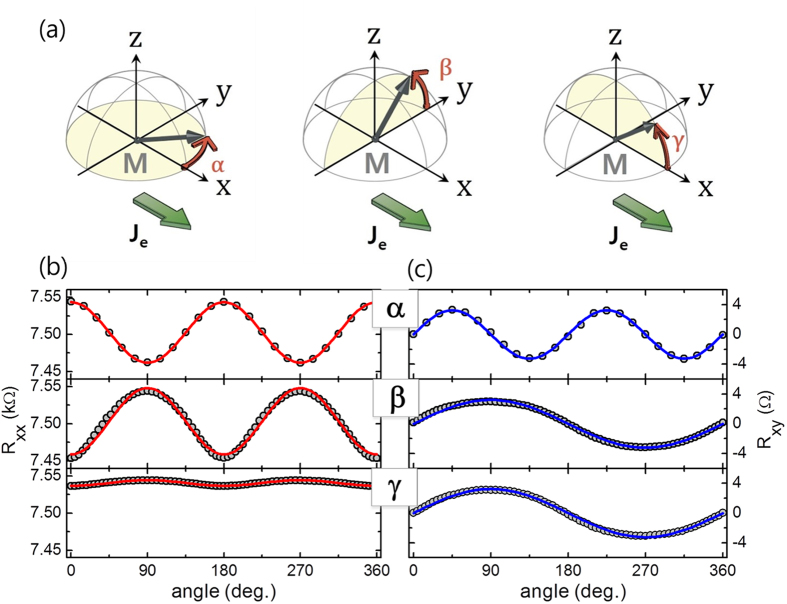
Angular dependence of the magnetoresistance. (**a**) Schematic of the MR measurement using the rotating sample in a strong magnetic field, of which angles are designated as α, β, and γ, respectively. *R*_*xx*_ (**b**) and *R*_*xy*_ (**c**) as a function of the rotating angle α, β, and γ. The measurements were done by rotating samples in a magnetic field, 1.5 T for α-rotation and 8 T for β- and γ-rotation.

We first present the measurement of the longitudinal (*R*_*xx*_) and transverse resistances (*R*_*xy*_) of a W(5 nm)/CoFeB(1.2 nm)/MgO(1.6 nm) sample as a function of the in-plane magnetic fields of *H*_*x*_ and *H*_*y*_ in Fig. 1(b,c), respectively. As the sample has perpendicular magnetic anisotropy, the in-plane magnetic fields rotate the magnetization from an out-of-plane (*z*-direction) to an in-plane (*x*- or *y*- direction). We apply the in-plane magnetic field up to 1.5 T, which is larger than the anisotropy field (~1T) of the sample. As shown in Fig. 1(b), *R*_*xx*_ is strongly dependent on the direction of the magnetic field. First, we observe that the variation of *R*_*xx*_ is nearly constant (~0.1%) for *H*_*x*_, when applied along the current direction. The application of *H*_*x*_ rotates 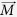 on the *x-z* plane such that the angle between 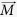 and the current *I*_*x*_ varies from 90^o^ to zero. Therefore, the insensitivity of *R*_*xx*_ to *H*_*x*_ demonstrates a negligible conventional AMR effect in this sample. Note that crystalline contribution of the AMR effect^23^,^24^ is ignored due to a thin CoFeB with an amorphous-like structure. In contrast, *R*_*xx*_ is gradually reduced with the application of *H*_*y*_, for which 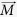 is always perpendicular to *I*_*x*_. In this field geometry, *R*_*xx*_ is sensitive to the relative angle of 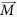 with respect to the *y*-direction. This magnetoresistance can be attributed to SMR^8^, in which the SHE-induced spins pointing in the *y*-direction of NM interact with the local magnetic moment of FM depending on the relative angle between them. For 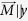, the accumulated spins are maximally reflected at the NM/FM interfaces^25^ and are transferred to an additional charge current via ISHE, resulting in lower resistance. We note that the SMR in the W/CoFeB/MgO sample is ~1.15%, which is approximately one order of magnitude greater than those reported for Pt/YIG structures^8^,^10^,^11^,^12^,^13^. This clearly confirms that SMR is also present in metallic FM/NM structures, in which charge current can flow through both FM and NM layers. This suggests that the current SMR model should be modified by including metallic FM. On the other hand, the transverse resistance *R*_*xy*_ decreases as the in-plane field is increased, irrespective of the field direction. This indicates that *R*_*xy*_ is dominated by the anomalous Hall effect (AHE)^26^ such that the SMR contribution to *R*_*xy*_ is negligibly small, which, however is only valid for the magnetic field of *H*_*x*_ or *H*_*y*_. Note that the sign of AHE in W/CoFeB/MgO samples is identical to those of Ta/CoFeB/MgO and Pt/Co/AlOx samples as shown in Fig. S1 in the supplementary information. The general feature of the contribution of SMR to *R*_*xy*_, shown in Fig. 2, will be discussed.

In order to confirm the angular dependence of *R*_*xx*_ and *R*_*xy*_, we repeat the measurement while rotating the samples on three major planes, i.e., the *x-y*, *y-z*, and *x-z* planes, under a strong magnetic field. As shown in Fig. 2(a), the typical angles of each plane are denoted as α, β, and γ, respectively. We note that the magnetic field of 1.5T (8T) for α (β and γ) rotation is larger than the anisotropy field of ~1T. Figure 2(b) shows that *R*_*xx*_ varies significantly with α and β, but remains nearly constant with γ. The longitudinal resistivity (*ρ*_xx_) in the FM/NM bilayer structure can be expressed^9^ as


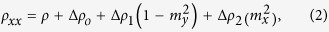


where *ρ* is the intrinsic electric resistivity, Δ*ρ*_o_ is the resistivity reduced by the spin-orbit interaction, and Δ*ρ*_1_ (Δ*ρ*_2_) is the change in the resistivity owing to SMR (AMR). The *x* and *y* components of the magnetization (*m*_*x*_ and *m*_*y*_) are equivalent to *cosγ* and *cosβ*, respectively. By fitting the angular dependence curves (Fig. 2(b)) using Eq. (1), we obtain a Δ*ρ*_1_ value of ~3.4 μΩcm (Δ*R*~85Ω in the measurement graph) and a Δ*ρ*_2_ value of 0.32 μΩcm (Δ*R*~8Ω). Therefore, in the W/CoFeB/MgO samples, SMR is much more dominant than AMR.

On the other hand, *R*_*xy*_ depends on the rotating angles in all three directions (Fig. 2(c)). The dependence of *R*_*xy*_ on β and γ is due to the AHE, in which *R*_*xy*_ is gradually reduced as 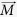 rotates toward the in-plane direction, whereas the variation of *R*_*xy*_ with α is attributed to the planar Hall effect (PHE)^27^. We note that PHE is normally a transverse component of AMR (i.e., transverse AMR). However in this sample, AMR is negligible; hence, PHE can be attributed to the transverse SMR, This can explain the large PHE value observed in similar structures^28^, which is comparable to that of the AHE.

**Figure 3:**
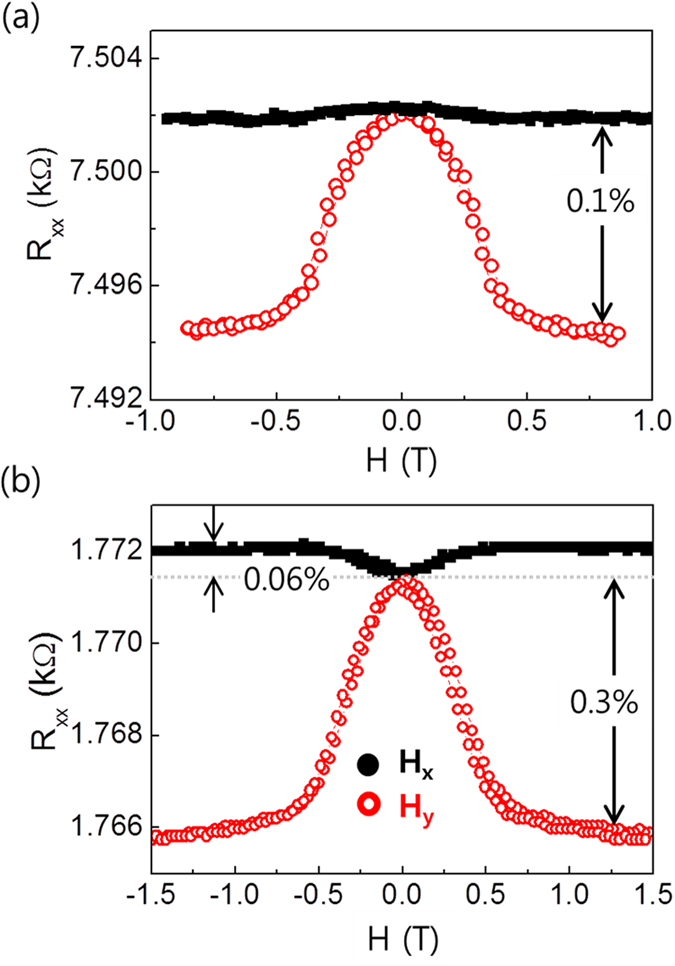
Longitudinal magnetoresistance (*R*_*xx*_) for different NM underlayer. (**a**) Ta(5 nm)/CoFeB(1 nm)/MgO(1.6 nm) and (**b**) Pt(3 nm)/Co(1 nm)/AlOx(1.5 nm) samples. The black solid (red open) circles represent the data for *H*_*x*_ (*H*_*y*_).

We compare the *R*_*xx*_ values of the W/CoFeB/MgO samples with those of samples with different underlayers. Figure 3 shows the *R*_*xx*_ values of Ta/CoFeB/MgO sample (a) and Pt/Co/AlOx sample (b) as a function of two different magnetic fields of *H*_*x*_ (black solid symbols, 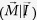 and *H*_*y*_ (red open symbols, 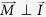). Both samples show behavior similar behavior to that of the W/CoFeB/MgO samples, i.e., a stronger dependence of *R*_*xx*_ on *H*_*y*_ rather than *H*_*x*_, demonstrating that SMR is the dominant magneto-transport mechanism in these NM/FM/oxide structures. However, the magnitude of the SMR of the sample with Ta or Pt is considerably smaller than that of the W/CoFeB/MgO structure, despite the fact that it is still much larger than those of the Pt/YIG samples^8^,^10^,^11^,^12^,^13^. Because the SMR mechanism is known to be a combination of the SHE and ISHE, the larger SMR can be explained by a larger spin Hall angle of W as compared to that of Ta or Pt, which is consistent with the values reported in the literature^4^,^21^,^22^,^29^,^30^. Given that the SMR is proportional to the square of spin Hall angle^9^, the relative magnitude of the SMR indicates that the spin Hall angle of W is approximately three (two) times larger than that of the sample with Ta (Pt).

### Thickness dependence of the spin Hall magnetoresistance

**Figure 4:**
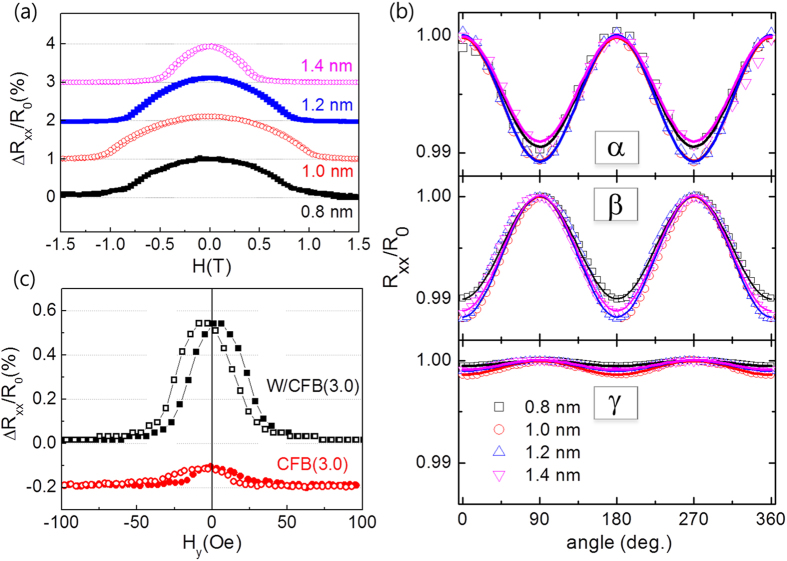
Dependence of the magnetoresistance on the CoFeB thickness. The MR for the sample W(5 nm)/CoFeB(*t*)/MgO(1.6 nm), where *t* varies from 0.8 to 1.4 nm using field sweep (**a**) and rotating (**b**) measurement. (**c**) *R*_*xx*_ vs *H*_*y*_ curves for the samples W(5 nm)/CoFeB(3 nm) and CoFeB(3 nm). Both samples show the in-plane magnetic anisotropy.

For a better understanding of the SMR of W/CoFeB/MgO samples, we investigate the dependence of SMR on the thicknesses of the W and CoFeB layers. We initially examine the effect of the CoFeB thickness on SMR. Figure 4(a) shows *R*_*xx*_ as a function of a transverse field, *H*_*y*_ for samples with different CoFeB thicknesses ranging from 0.8 to 1.4 nm, in which perpendicular magnetic anisotropy can persist. We find that *R*_*xx*_ normalized by the resistance at *H*_*y*_ = 1.5 T (*R*_0_) does not vary much with the CoFeB thickness. The similar angular dependence levels of these four samples, as shown in Fig. 4(b), confirms that *R*_*xx*_ does not significantly rely on the CoFeB thickness. To verify the role of the W layer, we compare samples of thicker CoFeB (3.0 nm) with and without a W layer, both of which showing in-plane magnetic anisotropy. Figure 4(c) shows *R*_*xx*_ as a function of *H*_*y*_. We note that the magnetization of both samples is aligned in the *x*-direction without a magnetic field due to the shape anisotropy of the Hall bar structure. The MR is considerably larger for the sample with W underlayer. The results shown in Fig. 4 clearly demonstrate that the W layer plays a key role in the observed SMR.

**Figure 5:**
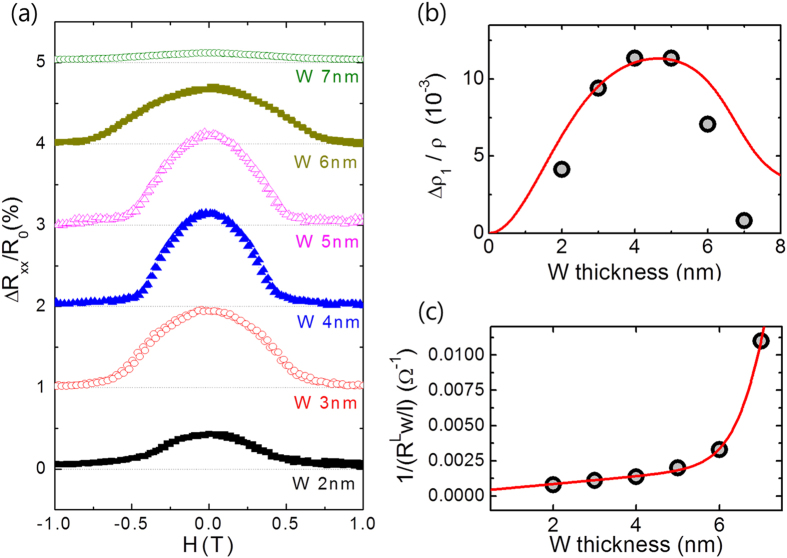
Dependence of the magnetoresistance on the W thickness. (**a**) MR vs *H*_*y*_ for the sample W(*t*)/CoFeB(1 nm)/MgO(1.6 nm), where *t* varies from 2 to 7 nm. (**b**) SMR as a function of the W thickness together with a theoretical fitting curve. (**c**) 1/*R*_*xx*_ vs the W thickness, from which the resistivity of W is extracted.

Next we examine the dependence of SMR on the W thickness in W(2 ~ 7 nm)/CoFeB(1.0 nm)/MgO(1.6 nm) samples. As shown in Fig. 5(a), the normalized *R*_*xx*_ is the largest for a W value of 4 or 5 nm and is reduced for a thicker or thinner W layer. This strong thickness dependence supports the argument that MR in W/CoFeB/MgO structures is mainly influenced by the SHE in W. The SMR can be expressed by the equation^9^,^10^.


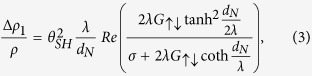


where *θ*_*SH*_, *λ*, and *d*_*N*_ are the spin Hall angle, the spin diffusion length, and the thickness of the NM layer, respectively. In addition, *σ* = *ρ*^*−1*^ is the conductivity and *Re G*_↑↓_ is real part of the spin mixing conductance with a unit of Ω^−1^m^−2^. According to Eq. (3), SMR decreases when the W layer is thinner than the spin diffusion length due to the reduced spin current caused by back reflection at the interface. On the other hand, for a thicker W layer, SMR is also reduced by a current shunting effect. With the fitting of the thickness dependence of the SMR and the resistivity to Eq. (3) (red line in Fig. 5(b)), a spin Hall angle of 0.21±0.01, and a spin diffusion length of 2.1±0.5 nm can be extracted. The spin mixing conductance of the W/CoFeB is priorly obtained to be 3.9 (±0.8) × 10^14^ Ω^−1^m^−2^ in time-resolved magneto-optical Kerr effect experiment. The details of the fitting procedure are described in the supplementary information 4. Note that the thickness dependent changes in resistivity extracted from Fig. 5(c) are considered for better fitting shown in Fig. 5(b). Moreover, as the crystallographic structure of a thicker W layer may differ from that of a thinner β-phase one^21^, the spin hall angle may also differ accordingly. This may lead to a slight discrepancy of the fitting data and the experimental data in the thicker W region (W > 5 nm), shown in Fig. 5(b) where, we assumed a constant spin hall angle.

### Correlation between the spin Hall magnetoresistance and the spin-orbit torque

**Figure 6:**
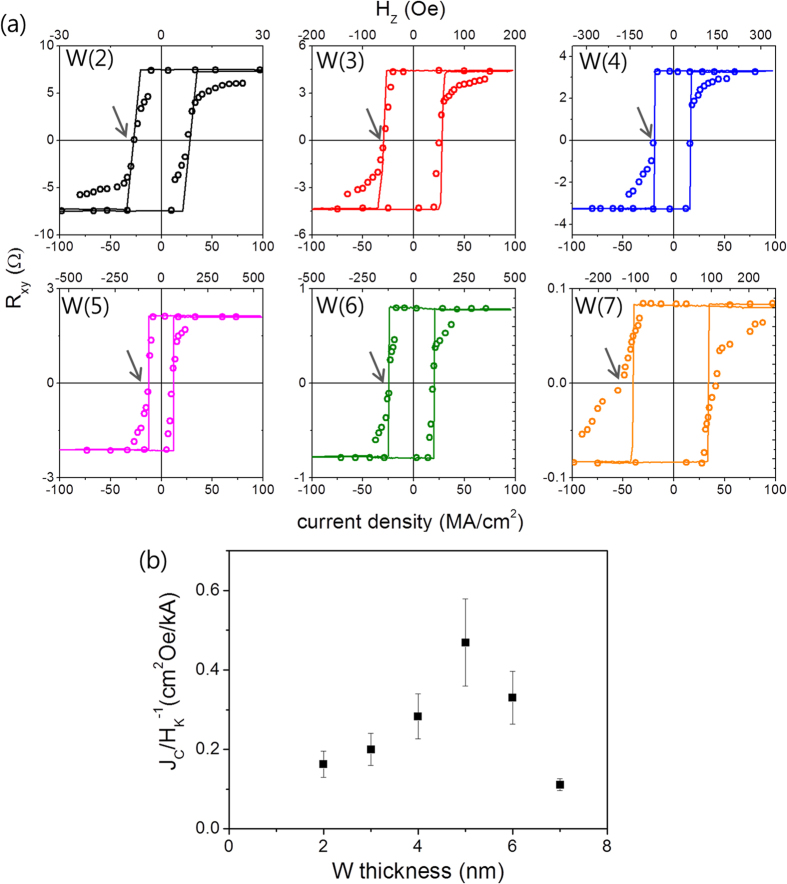
Switching experiments utilizing spin-orbit torque induced by in-plane current. (**a**) The magnetization direction detected by AHE measurement after each pulsed current of 10μs while sweeping current. The in-plane magnetic field *H*_*x*_ of 200 Oe is continuously applied during the measurement. Each line is independent AHE measurement as a function of *H*_z_, which is designated in the top axis. The arrows indicate the critical current density for magnetization switching. (**b**) The inverse ratio of critical current density (*J*_*c*_) to magnetic anisotropy (*H*_*k*_) as a function of the W thickness, which is corresponded to the SOT-switching efficiency or the magnitude of SOT.

Thus far, we have investigated the transport characteristics of W/CoFeB/MgO samples. Next, we examine an inverse effect of SMR, i.e., the in-plane current-induced spin-orbit torque (SOT). In order to evaluate the SOT magnitude, we perform a switching experiment using the same samples shown in Fig. 5. We first initialize the magnetization in the + *z* direction, and then sweep a pulsed current with a pulse width of 10 μs from a positive to a negative value, and vice versa, while maintaining a longitudinal magnetic field *H*_*x*_ of 200 Oe, which is necessary for deterministic switching^3^,^5^. After each current pulse, the magnetization direction is detected by measuring the AHE voltage. When the applied negative pulsed current exceeds a certain threshold, a reversal of the magnetization from* + z* to *–z* direction is observed. The AHE measured as a function of *H*_*z*_ is plotted as a line on the graph, indicating complete magnetization switching by the current-induced SOT. Note that a negative current and a positive *H*_*x*_ favor the *-z* direction of magnetization, which corresponds to the SOT with a negative spin Hall angle. We repeat the switching experiments for samples with various W thicknesses as shown in Fig. 6(a). We find that the critical current density (*J*_*c*_) for magnetization switching (marked by arrow) strongly depends on the W thickness. For example, switching can be done at a *J*_*c*_ value of ~11 MA/cm^2^ for samples with a W value of 5 nm, while it exceeds 42 MA/cm^2^ when W for such samples is 7 nm. In order to compare the SOT magnitude from the switching experiment, the relative amount of the critical current density with respect to magnetic anisotropy (*H*_*k*_) is plotted in Fig. 6(b), as the ratio of (*J*_*c*_/*H*_*k*_)^−1^ is a rough estimate of the SOT strength^31^. Here, *H*_*k*_ is obtained from the resistance vs. *H*_y_ curves in Fig. 5(a), where the resistance is saturated. This shows that the ratio (*J*_*c*_/*H*_*k*_)^−1^ reaches its maximum at 5 nm W, where SMR is also the largest (see Fig. 5(b)). Given that the ratio corresponds to the SOT efficiency, this finding indicates that the W thickness dependence of the SOT magnitude is identical to that of SMR, suggesting that the SMR and the SOT share the same physical origin of the SHE.

## Discussions

W/CoFeB/MgO structures show considerably greater SMR than structures with FM insulators or FM metal with other NM materials. This can be attributed to a large spin Hall angle of W as the SMR mainly originates from the SHE in NM. However, we note that there may be some contributions from the FM or FM/NM interface as well. The SMR reported in most studies which utilize Pt/YIG structures^8^,^10^,^11^,^12^,^13^ is approximately 0.01% ~ 0.1%, which is smaller than the result from the Pt/Co/AlOx structure as shown in Fig. 3(b). Assuming the same spin Hall angle of Pt, the large difference of the SMR depending on the FM material indicates that the spin Hall effect is not the sole origin of the SMR effect, but other contribution of FM or FM/NM interfaces^32^, for example, interfacial Rashba effect^33^,^34^ or magnetic proximity effect^35^ may be existent. The magnetic proximity effect is less likely in W-based samples because W is far from the Stoner instability. However, further study is required to clarify the origin of the SMR.

We demonstrate in this work that SMR experiments can be utilized to extract the spin Hall angles and spin diffusion lengths of the NM materials in the FM/NM structures, which are essential to interpret various spin transport phenomena related to spin-orbit coupling. Unlike other methods in which spin pumping (or excitation) is involved, this SMR measurement allows one to obtain these parameters with a simple electrical measurement.

Lastly, the similar trend of SMR and the magnetization switching efficiency induced by the spin-orbit torque (SOT) confirms the strong correlation between them. Since SOT has been intensively investigated given its high potential for device applications such as current-induced magnetization switching and domain wall motion with high speeds, a close examination of SMR can be very useful for understanding SOT physics.

## Methods

The sample structure of W/Co_32_Fe_48_B_20_(CoFeB)/MgO were grown on thermally oxidized silicon substrates by DC/RF magnetron sputtering under an Ar pressure of 3~10 mTorr. Here, the W and CoFeB thicknesses ranged from 2 to 7 nm and from 0.8 to 1.4 nm, respectively. The resistivity of CoFeB is ~330 μΩcm and that of W is ~370 μΩcm for thicknesses of less than 5 nm. An additional Ta (1 nm) capping layer on top of MgO (1.6 nm) was deposited to prevent contamination of the MgO layer. After the deposition, thin films were annealed at 250 °C for 30 min in vacuum condition (less than 10^−5^ Torr), which enhanced the perpendicular magnetic anisotropy. Hall bar patterned devices for transport measurements (Fig. 1(a)) were fabricated using photo-lithography and Ar ion milling. The length and width of the Hall bar structure were 75 μm and 5 μm, respectively. The longitudinal (*R*_*xx*_) and transverse resistance (*R*_*xy*_) were measured simultaneously using a DC current of 50 μA while sweeping the in-plane magnetic field or rotating the sample on the *x-y*, *y-z*, or *x-z* planes in a magnetic field much larger than the anisotropy field. A magnetic field of 8 T is applied for the rotating measurement of the β or γ direction, as this value should overcome the perpendicular magnetic anisotropy of ~1 T to align the magnetization parallel to the field direction. On the other hand, a magnetic field of 1.5 T for the rotation of α direction is enough to saturate the magnetization in the plane. The current-induced spin-orbit torque values are studied by performing switching experiments. The magnetization is detected by measuring the AHE voltage after each current pulse with a 1 μs width with the application of an in-plane magnetic field (*H*_*x*_) of 200 Oe, parallel to the current direction, for deterministic switching. This is compared with the AHE with a perpendicular magnetic field of *H*_z_. All measurements are done at room temperature.

## Additional Information

**How to cite this article**: Cho, S. *et al*. Large spin Hall magnetoresistance and its correlation to the spin-orbit torque in W/CoFeB/MgO structures. *Sci. Rep*. **5**, 14668; doi: 10.1038/srep14668 (2015).

## Supplementary Material

Supplementary Information
