# Supplementary material for: Large spin Hall magnetoresistance and its correlation to the spin-orbit torque in W/CoFeB/MgO structures
Source: Sci Rep. 2015 Oct 1;5:14668. doi: 10.1038/srep14668 (PMC4589767; doi:10.1038/srep14668)
Supplement: Supplementary Information [file srep14668-s1.pdf]

Supplementary Information

Large spin Hall magnetoresistance and its correlation to the spin-orbit torque in W/CoFeB/MgO structures

Soonha Cho[1], Seung-heon Chris Baek[1,2], Kyeong-Dong Lee[1], Younghun Jo[3], and Byong-Guk Park[1,*]

[1] Department of Materials Science and Engineering, KAIST, Daejeon 305-701, Korea

[2] Department of Electrical Engineering, KAIST, Daejeon 305-701, Korea

[3] Division of Scientific Instrumentation KBSI, Daejeon 305-806, Korea

* Corresponding email: bgpark@kaist.ac.kr (B.-G. Park)

1. Comparison of AHE with different material systems

We examine a sign of AHE in W/CoFeB/MgO samples by comparing with AHE of widely-studied structures of Ta/CoFeB/MgO and Pt/Co/AlOx samples measured using the same electrical connection as Fig. 1 (a) of the main text. As shown in Fig. S1, the results demonstrate that a sign of AHE in W/CoFeB/MgO sample is identical to that of Ta/CoFeB/MgO or Pt/Co/AlOx structures.

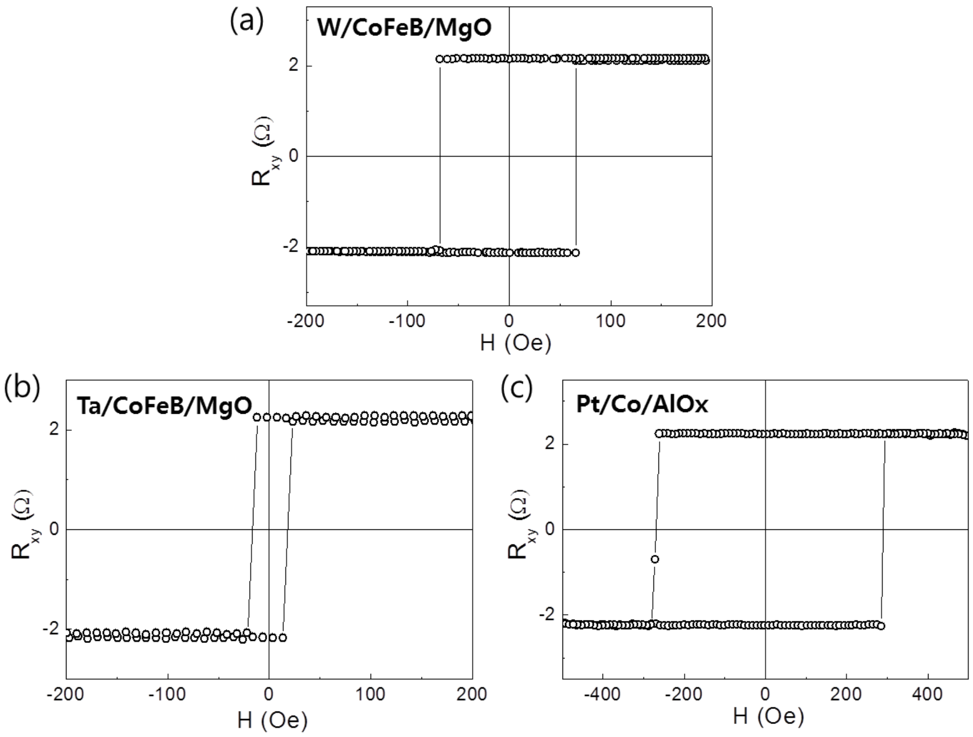

Figure S1| Anomalous Hall effect (AHE) in various perpendicularly-magnetized structures of (a) W(5 nm)/CoFeB(1 nm)/MgO, (b) Ta(5 nm)/CoFeB(1 nm)/MgO, and (c) Pt(3 nm)/Co(1 nm)/AlOx, which are measured using the same electrical connections as Fig. 1(a) of the main text.

2. Current dependence of spin Hall magnetoresistance in W/CoFeB/MgO

The dependence of SMR on current density is studied by measuring SMR with various currents ranging from 10 μA to 70 μA. Fig. S2 shows the variation of longitudinal resistance ($\Delta R_{xx}/R_0$) and transverse resistance (R_{xy}) in W(5 nm)/CoFeB(1.2 nm)/MgO sample as a function of transverse magnetic field H_y, which are almost the same regardless of current level. This demonstrates that the SMR is not affected by the magnitude of the applied current, at least within the range used in the measurement shown in Fig S2.

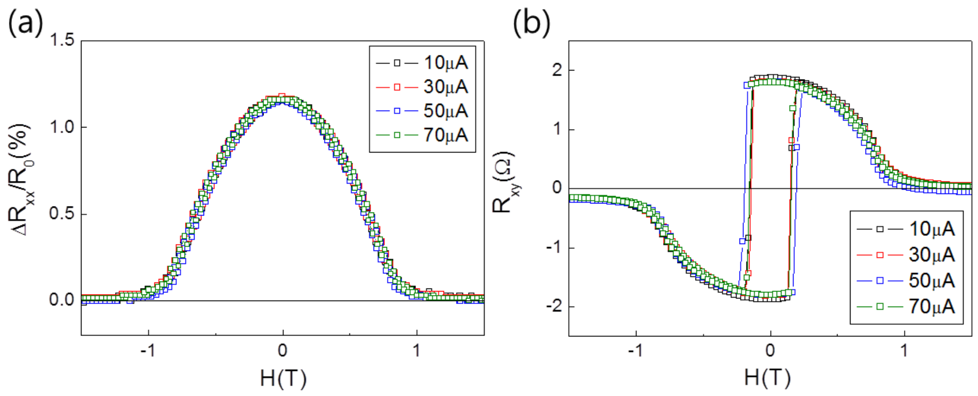

Figure S2| Spin Hall magnetoresistance (SMR) with different currents ranging from 10 μA to 70 μA. (a) Longitudinal resistance ($\Delta R_{xx}/R_0$) and (b) transverse resistance (R_{xy}) variation are measured as a function of transverse magnetic field H_y in the W(5 nm)/CoFeB(1.2 nm)/MgO(1.6 nm) sample, which is the same sample as one in Fig 1 (b, c) of the main text.

3. Angular dependence of magnetoresistance in Pt/Co/AlOx

The angular dependence of longitudinal and transverse resistance (R_{xx} and R_{xy}) of the Pt/Co/AlOx sample is also measured using a similar method as Fig. 2 in the main text. Figure S3 shows the resistance variation of the rotating samples in three major planes, *x*-*y*, *y*-*z*, and *x*-*z* plane as a function of the representative angles of each plane, α, β, and γ, respectively. A magnetic field of 1.5 T is applied for all three rotations, which is ~ 3 times larger than the anisotropy field of ~0.5 T for Pt/Co/AlOx sample. Like the W/CoFeB/MgO samples in the main text, the variation of the R_{xx} is much larger for β rotation than γ one. This corroborates that SMR is a dominant transport mechanism in the Pt/Co/AlOx structures as well. We note that AMR effect in Pt/Co/AlOx sample is a bit greater than that in W/CoFeB/MgO samples. Figures 1(b) and 3(b) show the longitudinal resistance variation as a function of H_x and H_y which correspond to AMR and SMR effect, respectively. We compared AMR/SMR ratio in both W/CoFeB/MgO and Pt/Co/AlOx samples and the numbers were 0.05 to 0.2 respectively, where Pt/Co/AlOx sample showed 4 times larger AMR contribution than that of W/CoFeB/MgO sample. Furthermore, we presume that the greater AMR effect in Pt/Co/AlOx sample might be due to the proximity effect in Pt. However, further study is required to fully explain this phenomenon. On the other hand, the angular dependence of the R_{xy} shown in Fig S3 (b, d, f) is very similar to that of the W/CoFeB/MgO samples as shown in Fig 2(c) in the main text.

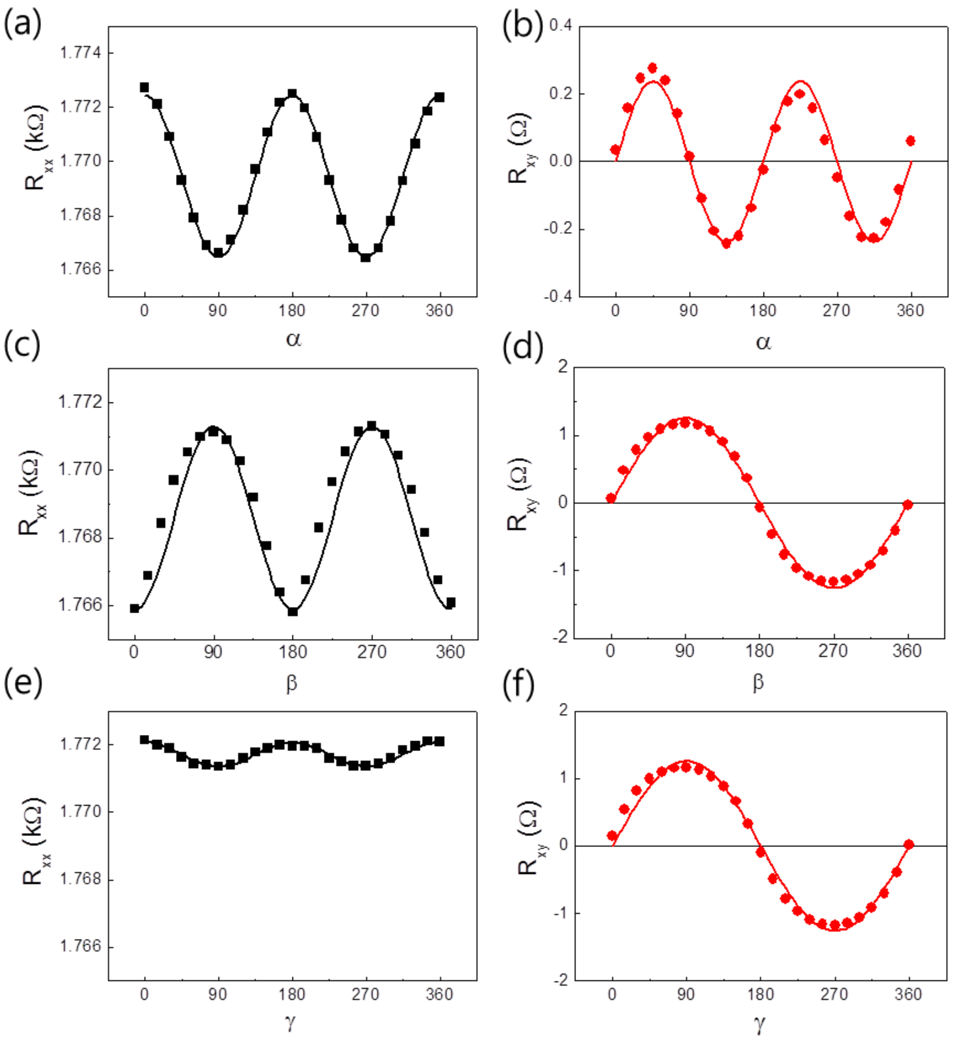

Figure S3| Angular dependence of longitudinal resistance R_{xx} (a, c, e) and transverse resistance R_{xy} (b, d, f) in Pt(3 nm)/Co (1 nm)/AlOx (1.6 nm) sample as a function of the rotating angle α, β, and γ, respectively. The measurements were done by rotating samples in a magnetic field of 1.5 T for three angles.

4. Extraction of spin Hall angle, spin diffusion length, and spin mixing conductance

Spin Hall angle, spin diffusion length and spin mixing conductance can be extracted from the fit of the Eq. (3) to the data in Fig. 5 (b) of the main text. However, it is not easy to extract the three unknown values from a single fit. Nevertheless, among the three the spin diffusion length governs the thickness dependence of SMR, so it can be obtained by the peak position of the SMR vs. W thickness curve. We used the thickness dependent resistivity of W, as shown in Fig. S4(a), which is obtained from Fig. 5(c) in the main text. As shown in the fitting curve of Eq. (3) of Fig. S4(b), especially when W thickness is larger than 5 nm, the fitting red curve considering these changes in resistivity shows better match with the experimental data than the black curve, where constant resistivity of W (~370μΩcm) is used. On the other hand, spin Hall angle and spin mixing conductance which determines the magnitude of the SMR are strongly correlated [S1]. Figure S4(c) shows a variation of extracted spin Hall angles from W thickness dependence of the SMR, depending on the magnitude of spin mixing conductance. In order to obtain a proper value of spin Hall angle, we perform independent experiment of time-resolved magneto-optical Kerr effect (TRMOKE), from which the spin pumping can be measured [S2, S3], and spin mixing conductance can be estimated [S4, S5].

Figure S5(a) shows a schematic TRMOKE measurement. An external magnetic field H is applied at an angle of 15° with respect to the sample plane to suppress domain formation [S3], and a pump laser pulse ($\Delta t = 0$) is then used to induce spin precession of the sample, since the laser heating changes the anisotropy K_{eff} and magnetization M, thereby altering the equilibrium M-orientation θ. After the heating ($\Delta t > 0$), M starts to precess around its effective equilibrium, and dissipates the excited energy via Gilbert damping and the

restoration of K_{eff} and M. The polar component (M_z) of the precession is measured from the change in the polarization of the reflected probe beam with delay time Δt using a balanced detection technique, due to high sensitivity to the out-of-plane component of the magnetization. The probe (pump) beam has a central wavelength of 400 (800) nm, an average power of ~ 40 μW (6 mW), and a repetition rate of 82 MHz.

The observed TRMOKE signal is shown in Fig. S5(b) for W(5 nm)/CoFeB(0.8, 1, 3 nm) bilayer and a single CoFeB (3 nm) sample. Here, a thick 5-nm W layer is used for minimizing backflow effect to simplify the analysis [S5, S6], and H = 1.86 T. To analyze the data, TRMOKE traces are fitted with the following phenomenological fitting formula:[S3]

$$-\frac{\Delta M_z}{M_z} = \mathrm{A}_1 + \mathrm{A}_2 e^{-\frac{\Delta t}{\tau_e}} + \frac{A_3}{\sqrt{1+\frac{\Delta t}{t_0}}} + \mathrm{A}_4 e^{-\frac{\Delta t}{\tau_d}} \sin(2\pi f \Delta t + \varphi), \qquad \text{(S1)}$$

where Δt is the delay time between pump and probe beam, and A_1 is the non-magnetic background. The second term represents relaxation of the electron temperature with an equilibration time τ_e. The third term expresses one dimensional heat diffusion with the absorption temporal profile t_0. Precessional magnetization motion is described in the last term. Examples of fitted curves are depicted in Figs. S5, where the lines denote the fits. From the fits, the damping parameter can be obtained by the following relation:

$$\alpha_{eff} = (2\pi f \tau_d)^{-1} \qquad \text{(S2)}$$

where α_{eff} is the effective Gilbert damping constant, f the precession frequency, and τ_d the relaxation time of the precession.

The spin mixing conductance is related to the change of the damping constant by the attachment of a non-magnetic layer, which can be extracted using an equation [S4, S5]

$$g_{\uparrow\downarrow} = \frac{4\pi M_s t_F}{g\mu_B}(\alpha_{W/CoFeB} - \alpha_{CoFeB}), \quad \text{(S3)}$$

where μ_B, g, M_s and t_F is Bohr magnetron, g factor, saturation magnetization and thickness of ferromagnetic layer of CoFeB. Before directly applying the measured α_{eff} to obtain spin pumping contribution of $\Delta\alpha = \alpha_{W/CoFeB}$ - α_{CoFeB}, we need to verify the elimination of the inhomogeneous effect like two magnon scattering [S3, S5] and to confirm the spin diffusion length to apply the simple Eq. (S3).

At first, we verified the absence of the inhomogeneous effect by varying the resonance precession frequency as shown in Fig. S6. Here, when the external field H > 1.3 T, α_{eff} nearly converges to a single value, which implies the exclusion of inhomogeneous effect [S7].

Hence, by applying H = 1.86 T > 1.3 T, α_{eff} is measured with W(t = 1, 3, 5, 7 nm)/CoFeB(3 nm) to identify the spin diffusion length λ of W, as shown in Fig. S7. Here, we can obtain λ of 2.3 ± 0.9 nm by utilizing backflow contribution: $1 - e^{\frac{-2t}{\lambda}}$ [S5]. We would like to note that λ is consistent to the value obtained from SMR fitting. This means that Eq. (S3) is adequately valid when we use 5-nm W to estimate $g_{\uparrow\downarrow}$ [S6].

Figure S8 shows the fit to extract spin mixing conductance from the data of Fig. S6. Using the Eq. (S3), spin mixing conductance of the CoFeB/W interface is obtained to be $g_{\uparrow\downarrow}$~1.0 (±0.2)×10^{19} m^{-2} or $G_{\uparrow\downarrow}$~3.9 (±0.8)×10^{14} $\Omega^{-1}\cdot m^{-2}$ by using the relation $G_{\uparrow\downarrow} = (e^2/h)g_{\uparrow\downarrow}$ [S8].

This $g_{\uparrow\downarrow}$ value as well as spin diffusion length and Gilbert damping constant of 3-nm CoFeB (~0.0051 ± 0.0007) are in similar order of those values in recent reports with Pd [S5], Pt [S6], W [S9], and 3-nm CoFeB [S10, S11]. This allows one to determine spin Hall angle of 0.21 (±0.01) as indicated in Fig. S4(c).

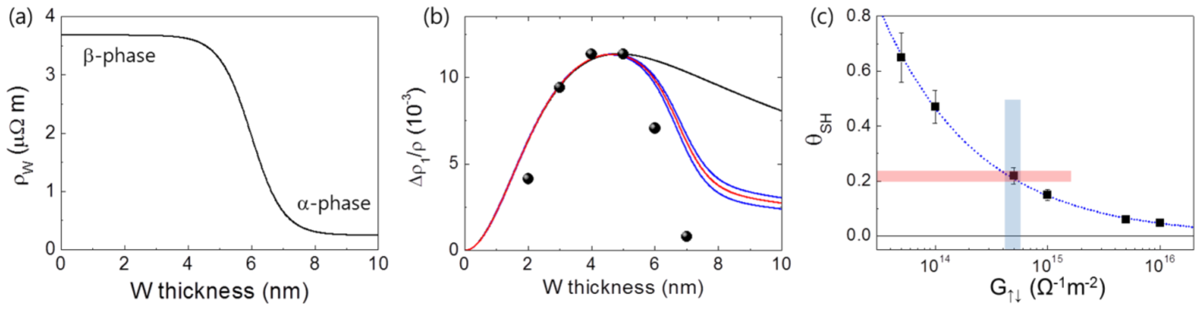

Figure S4| (a) Thickness dependent resistivity ρ_W of W. (b) Red curve shows the SMR value fit of Eq. (3), taking thickness dependent resistivity into consideration, as compared with black curve fit with constant resistivity. Additional blue curves with $G_{\uparrow\downarrow}$= 3.9 ± 0.8 (10^{14} $\Omega^{-1}\cdot m^{-2}$) are depicted to show the effect of $G_{\uparrow\downarrow}$ variation. (c) Correlation between spin Hall angle and spin mixing conductance, which extracted from the fit of Eq. 3 in the main text to the W thickness dependence of SMR value of Fig. 5(b) in the main text.

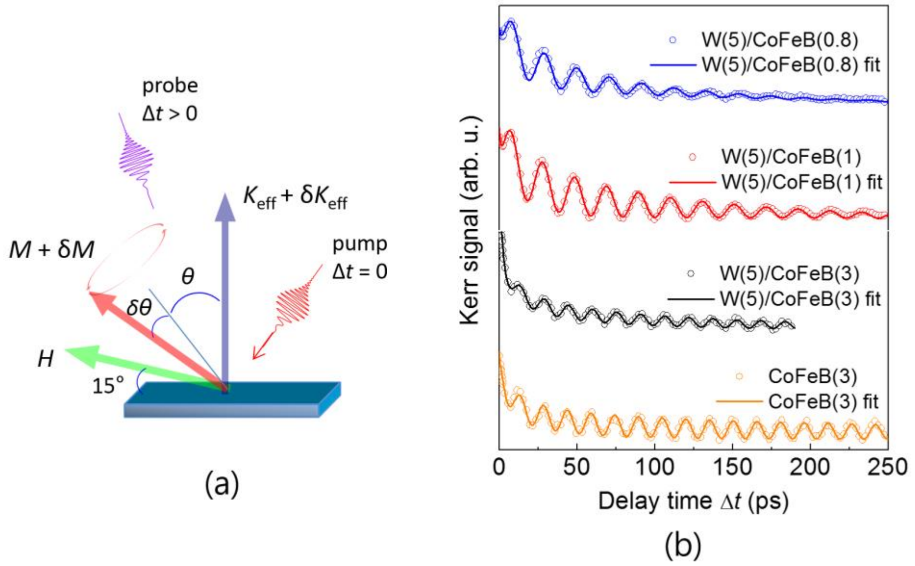

Figure S5| Time-resolved magneto-optical Kerr effect (TRMOKE). (a) Schematic TRMOKE measurement and the excitation process. (b) TRMOKE signal in CoFeB (3 nm) and W(5 nm)/CoFeB(0.8, 1, 3 nm) samples, when H = 1.86 T.

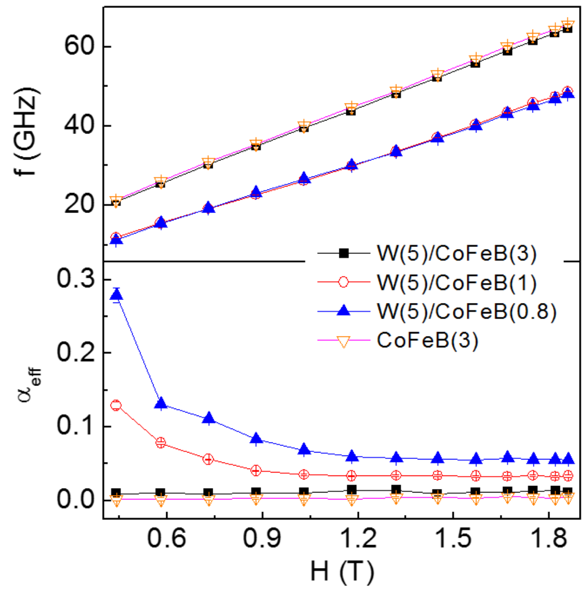

Figure S6| Resonance frequency *f* and the effective Gilbert damping constant α_{eff}, which are obtained from Fig. S5, and Eqs. (S1) and (S2).

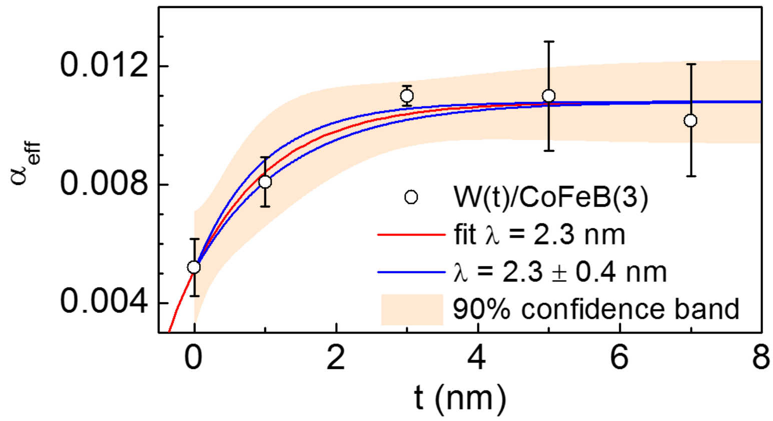

Figure S7| α_{eff} as a function of W thickness. Shaded area represents a band of 90% confidence level of the fitting. Additional guide lines with $\lambda = 2.3 \pm 0.4$ nm are depicted to show the effect of λ variation with respect to the data error bar of α_{eff}.

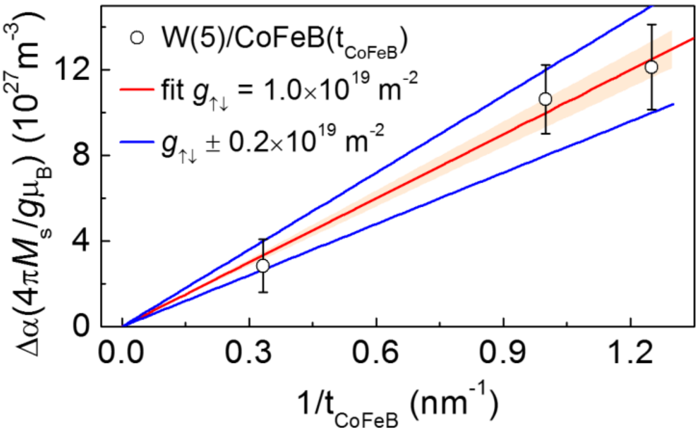

Figure S8| Spin pumping contribution, Δα as a function of $1/t_{\text{CoFeB}}$. Shaded area of fitting confidence level of 90% is smaller than the error bar of data point. Therefore, we estimate the error of the slope $g_{\uparrow\downarrow}$ to cover the error bar of data point as depicted in blue line.

References

S1. Vlietstra, N., Shan, J., Castel, V., van Wees, B.J. & Youssef, J.B. Spin-Hall magnetoresistance in platinum on yttrium iron garnet: Dependence on platinum thickness and in-plane/out-of-plane magnetization. *Phys. Rev. B* **87**, 184421 (2013).

S2. Urban, R., Woltersdorf, G. and Heinrich, B. Gilbert Damping in Single and Multilayer Ultrathin Films: Role of Interfaces in Nonlocal Spin Dynamics. *Phys. Rev. Lett.* **87**, 217204 (2001).

S3. Schellekens, A. J. *et al.* Determining the Gilbert damping in perpendicularly magnetized Pt/Co/AlOx films. *Appl. Phys. Lett.* **102**, 082405 (2013).

S4. Mosendz, O. *et al.* Quantifying Spin Hall Angles from Spin Pumping: Experiments and Theory. *Phys. Rev. Lett.* **104**, 046601 (2010).

S5. Shaw, J. M., Nembach, H. T. & Silva, T. J. Determination of spin pumping as a source of linewidth in sputtered $Co_{90}Fe_{10}$/Pd multilayers by use of broadband ferromagnetic resonance spectroscopy. *Phys. Rev. B* **85**, 054412 (2012).

S6. Jiao, H. & Bauer, G. E. W. Spin Backflow and ac Voltage Generation by Spin Pumping and the Inverse Spin Hall Effect. *Phys. Rev. Lett.* **110**, 217602 (2013).

S7. Song, H.-S. *et al.* Observation of the intrinsic Gilbert damping constant in Co/Ni multilayers independent of the stack number with perpendicular anisotropy. *Appl. Phys. Lett.* **102**, 102401 (2013).

S8. Tserkovnyak, Y., Brataas, A., Bauer, G. E. W. & Halperin, B. I. Nonlocal Magnetization Dynamics in Ferromagnetic Hybrid Nanostructures. *Rev. Mod. Phys.* **77**, 1375 (2005).

S9. Kim, J., Sheng, P., Takahashi, S., Mitani, S. & Hayashi, M. Giant spin Hall magnetoresistance in metallic bilayers. *arXiv*:1503.08903 (2015).

S10. Ikeda, S. *et al.* A perpendicular-anisotropy CoFeB-MgO magnetic tunnel junction. *Nat. Mater.* **9**, 721 (2010).

S11. Iihama, S. *et al.* Gilbert damping constants of Ta/CoFeB/MgO(Ta) thin films measured by optical detection of precessional magnetization dynamics. *Phys. Rev. B* **89**, 174416 (2014).
